# Exploring Different Management Modalities of Nonsyndromic Craniosynostosis

**DOI:** 10.7759/cureus.60831

**Published:** 2024-05-22

**Authors:** Bader M Al-Murad, Mohammed A Radwan, Ibrahim A Zaki, Mohammed M Soliman, Eatedal M AL-Shareef, Aseel M Gaban, Yara M Al-Mukhlifi, Fatma Z Kefi

**Affiliations:** 1 Medical School, Batterjee Medical College, Jeddah, SAU; 2 General Practice of Pediatrics, Batterjee Medical College, Jeddah, SAU; 3 Medicine School, Batterjee Medical College, Jeddah, SAU; 4 Medical School, Tabuk College, Tabuk, SAU; 5 Medical School, King Saud Bin Abdualziz University for Health and Sciences, Riyadh, SAU; 6 Medical School, Batterjee Medical College, Jeddah , SAU

**Keywords:** craniosynostosis surgery, review article, surgical management, conservative management, non-syndromic craniosynostosis

## Abstract

Craniosynostosis is an atypical skull shape characterized by the premature fusion of cranial sutures. It is one of the most common congenital anomalies encountered by craniofacial surgeons, with a prevalence of one in every 2000-2500 births. It is classified into two main types: syndromic and nonsyndromic. In syndromic, the patient presents with other abnormalities involving the trunk, face, or extremities. While in nonsyndromic the only anomy is the premature fusion, which usually involves one suture; the most common subtypes are unicoronal, sagittal, bicoronal, metopic, and lambdoid. As a consequence, premature fusion before its natural time restricts the space for the brain to grow, increases intracranial pressure, causes damage to the brain tissue, and affects the development of the child. This review comprehensively provides a detailed overview of nonsyndromic craniosynostosis and aims to highlight the importance of early and accurate diagnosis, and determining the most suitable intervention, whether surgical or conservative modalities. The optimal treatment approach produces the most favorable aesthetic and functional outcomes.

## Introduction and background

Craniosynostosis is defined as an atypical skull shape brought about by the premature fusion of cranial sutures [1]. It is one of the most prevalent congenital anomalies presenting to craniofacial surgeons, occurring in one in every 2000-2500 births [2,3]. Craniosynostosis involves two main types: syndromic and nonsyndromic. The syndromic form presents with other abnormalities of the trunk, face, or extremities and falls beyond the scope of the article. In the nonsyndromic kind, premature fusion is the only anomaly, and usually involves one suture; the most common subtypes being unicoronal, sagittal, bicoronal, metopic, and lambdoid [4]. The premature fusion restricts the space available for brain growth, elevating intracranial pressure, damaging brain tissue, and negatively impacting the general development of the child [1]. Early and accurate diagnostic evaluation of craniosynostosis is of great importance in determining the type of intervention required, whether surgical or conservative modalities, as prompt intervention produces the most favorable aesthetic and functional results [5].

Nonsyndromic craniosynostosis is considered to possess a multifactorial etiology, including environmental and genetic factors [6]. A precise genetic etiology for the disease is not completely understood; however, the condition has been found to correlate with more than 50 genes [7,8]. Among those are mutations involving TWIST1, EFNB1, and fibroblast growth factor/FGF receptor (FGF/FGFR) pathway genes. Furthermore, in identifying the role of disease-modifying genes associated with craniosynostosis, a genome-wide association study (GWAS) has spotted susceptibility loci close to bone morphogenetic protein-2 (BMP-2) in sagittal nonsyndromic craniosynostosis [7]. Another GWAS performed a meta-analysis that suggested insightful results in implicating the BMP7 locus as a genetic risk factor for premature metopic suture closure [9]. Environmental risk factors include maternal diabetes, smoking, excessive coffee intake, and thyroid dysfunction. Pregnant women on certain medications, such as selective serotonin reuptake inhibitors (SSRIs) and clomiphene citrate, which are used to treat infertility, put their future newborns at an increased risk of developing craniosynostosis [7].

In 2019, the global number of children born with craniosynostosis was estimated to be 84,665, including 72,857 cases of nonsyndromic craniosynostosis. The recent estimated overall global prevalence of craniosynostosis, according to a large meta-analysis, is reported to be 5.9 out of every 10,000 live births. While nonsyndromic craniosynostosis is reported to have a prevalence of 5.2 out of every 10,000 live births [10]. Additionally, males outnumber females in sagittal craniosynostosis in a ratio of 4:1; however, in unilateral coronal craniosynostosis, females outnumber males in a ratio of 3:2 [4].

Accounting for about 15-20% of cases, the condition is syndromic and presents as part of a genetic disorder due to certain mutations or chromosomal abnormalities [11,12]. This form involves multiple cranial sutures, consistently co-occurs with other malformations of the face, skull, or extremities, and pertains strongly to complications such as hydrocephalus, auditory and visual defects, and intellectual disability [4,7]. On the other hand, most cases occur sporadically as an isolated pathology without an identifiable genetic etiology. This nonsyndromic type, often, involves a single suture only and lacks the complications mentioned earlier [4,12]. Although nonsyndromic craniosynostosis typically involves one suture, it could seldom entail multiple sutures and this is the complex type [13].

Additionally, craniosynostosis can be categorized according to the suture involved and the resulting skull shape [14]. Fusion of the sagittal suture is the most common and brings about a characteristic boat-shaped deformity known as ‘scaphocephaly’ [14,15]. The second most prevalent type involves the coronal suture [14]. It is mostly unilateral and produces a head that is anteriorly asymmetrical and is referred to as anterior plagiocephaly [4]. The less frequent, bicoronal synostosis produces ‘brachycephaly’ or a short head that is wide; it is important to note that unlike the unilateral type, bicoronal fusion is almost always syndromic [14,16]. ‘Trigonocephaly’ with the pathognomonic triangular-shaped narrow forehead is the third most common variant and is attributed to premature fusion of the metopic suture. The affection of the lambdoid suture is the least common and, if unilateral, brings about ‘posterior plagiocephaly’ [14,15]. In bilateral lambdoid synostosis, the entire occiput is flattened and widened, and both ears are displaced anteroinferiorly [17].

Positional or deformational plagiocephaly is an important differential diagnosis of posterior plagiocephaly that occurs due to prolonged pressure on the same area of the child’s head [1]. It has been shown that the downward skull tilt and inferior shift of the ear are the most reliable indicators of actual posterior plagiocephaly [14].

## Review

Anatomic and neurodevelopmental consequences

Whether single-suture craniosynostosis causes affected newborns to have increased intracranial pressure (ICP), hydrocephalus, or decreased intracranial volume is still up for debate. Some studies were conducted to investigate how these factors affected ICP. According to Sgouros et al., there is no difference in intracranial volume between the various kinds of craniosynostosis when it comes to certain procedures that can increase it to above-normal levels [18]. In contrast, no appreciable variations in intracranial volume were seen between the newborns in Hill et al.’s study on unicoronal craniosynostosis and the control group. However, one must consider the limitations of the study, such as its limited scope and absence of physiological measures such as intracranial pressure [19]. Consequently, there is no evidence that low intracranial volume and craniosynostosis are related. This could be because of compensatory growth of the skull at unaffected sites or the addition of bone at the exterior, not the interior [20].

The relationship between hydrocephalus and craniosynostosis is unclear. A study by Cinalli et al. examining more than 1700 cases of craniosynostosis discovered that the rate of hydrocephalus in newborns with nonsyndromic craniosynostosis necessitating the implantation of a ventriculoperitoneal shunt was just 0.28% [21]. This shunting rate is identical to what is seen in the average population [22]. Subdural-peritoneal shunts were also required for 0.6% of patients after surgery, most likely because of increased intracranial volume without corresponding changes in brain volume, which resulted in a buildup of extra CSF [21].

Even though there is no observed association with lower intracranial volume or hydrocephalus, newborns with nonsyndromic or single-suture craniosynostosis are at increased risk of developing intracranial hypertension. When Gault et al. looked at 66 babies with craniosynostosis, they discovered that 20% of them had elevated intracranial pressure [20]. According to Renier et al., 30% of children with single-suture synostosis had ICP [23]. The incidence of cerebral hypertension was found by Thompson et al. to be 15% in cases of single-suture and 24% in cases with nonsyndromic craniosynostosis [24]. Our results emphasize the significance of keeping an eye on ICP. However, there has not been much research done to determine how elevated ICP affects neurocognitive development, and no clear correlation has been found [25].

Along with its association with intracranial hypertension, premature fusion of cranial sutures is known to alter the morphology of the underlying brain. The authors showed that both cortical and subcortical structures of the central nervous system are dysmorphic in craniosynostosis [26]. Specifically, studies of brain morphology in cases of sagittal and unicoronal synostosis have demonstrated that changes in brain structure are found not only in regions of the brain adjacent to the fused suture but also in distant and subcortical regions [27]. Furthermore, these studies showed that despite surgical correction of skull shape, the brain tends to follow a growth pattern like that observed in patients with untreated craniosynostosis, indicating at least partially independent growth trajectories of the skull [28].

Craniosynostosis, not only causes intracranial hypertension but also modifies the shape of the brain. Aldridge and colleagues discovered anomalies in both cortical and subcortical systems [26]. Research on forms of craniosynostosis has shown anatomical alterations both close to and far from the fused sutures [27]. The brain tends to retain growth patterns seen in untreated instances even after surgical correction, indicating partially distinct growth trajectories for the skull and brain [28].

Recent studies have demonstrated the effects of nonsyndromic craniosynostosis on behavior, speech, and cognition. Nearly half of patients who were five years or older after surgery had developmental problems [29]. They noticed that following surgery, deficiencies in attention, language, and spatial ability persisted, most likely because of continuing morphological alterations in the brain. There are various cognitive and behavioral impairments linked to specific types of craniosynostosis. For instance, metopic synostosis has been linked to behavioral concerns, while sagittal synostosis has been linked to speech and language impairment [30]. According to Shipster et al. (2003), children with sagittal synostosis have high rates of cognitive and linguistic disability [31]. Research conducted by Kelleher et al. (2006) and Bottero et al. (1998) revealed higher rates of behavioral and cognitive problems in isolated metopic synostosis cases [32,33]. Nevertheless, trigonocephaly severity was not found to be correlated with behavioral or cognitive outcomes. Furthermore, it has been suggested that a child’s risk of neurodevelopmental delay or impairment is not considerably impacted by the scheduling of surgeries [34].

Diagnostic evaluation

History

It is crucial to take a comprehensive history from the child’s parents to determine the etiology of craniosynostosis. History should focus on maternal exposure to teratogenic medications during pregnancy, such as valproic acid, and a family history of genetic disorders or craniofacial abnormalities [1]. Furthermore, inquiring about prenatal history is paramount, as certain factors such as maternal diabetes, smoking, excessive coffee intake, and thyroid dysfunction are linked to a higher risk of craniosynostosis [7]. Additionally, it is important to investigate possible causes of intrauterine head compression, such as multifetal pregnancy or oligohydramnios. The history should also include a detailed timeline of the infant’s developmental milestones [1].

Physical

As the diagnosis of craniosynostosis is primarily clinical, physical examination is an absolute cornerstone in the approach to these patients. To begin with, it is crucial to carefully look for any congenital anomalies or facial dysmorphic features that suggest a case of syndromic synostosis [1]. Subsequently, examining the shape of the infant’s head is necessary to determine if premature fusion is present in the first place. In most nonsyndromic cases, this examination allows pinpointing of the affected suture; this is attributed to the fact that simple or single-suture fusion produces a characteristic head phenotype, as discussed earlier [14]. Furthermore, as previously stated, it is at this stage that if an asymmetrical posterior head is noted, it is crucial to differentiate between deformational plagiocephaly and true lambdoid synostosis, as management lines contrast markedly; conservative and surgical, respectively [35]. Once craniosynostosis is suspected clinically, the patient must undergo an ophthalmic examination to rule out ICP [14].

Investigation

Current rapid low-dose computed tomography (CT) scan with three-dimensional (3D) reconstruction is the golden standard initial imaging modality for craniosynostosis [3]. It allows for preoperative planning through assessment of the following: suture patency, anthropometric measurements, and possible brain parenchymal or ventricular abnormalities such as Arnold-Chiari malformation and agenesis of the corpus callosum [3,11]. If cerebral anomalies are apparent on CT scan, magnetic resonance imaging (MRI) can be utilized for better visualization [11]. When clinical examination findings point toward syndromic synostosis, genetic testing is indicated [1].

Management (Surgical and conservative modalities)

Surgical Modalities

Endoscopic-assisted strip craniectomy with postoperative helmet therapy: A minimally invasive surgical technique called endoscopy-assisted strip craniectomy is used to treat craniosynostosis. Using an endoscope to view the fused suture, a strip of bone is removed along with the suture to allow the skull to grow naturally [36]. Compared to open surgery, this technique has benefits like fewer incisions, less blood loss, shorter recovery periods, and possibly quicker healing [37]. It is especially helpful for newborns between the ages of 3 and 4 months old, as well as for cases with a single suture, such as sagittal or metopic sutures. Once the surgical site is accessed, the surgeon proceeds to expand the osteotomy, executes the suturectomy, and extracts the bone strip [36]. Spring assistance can be used to facilitate biparietal vault widening [38]. In cases of early presented trigonocephaly, a wedged strip suturectomy is carried out, and spring aid may be utilized to broaden the skull [36]. Some studies suggest that this method can be used for early presented coronal synostosis (3-4 months) [39]. A second procedure might be required after 4 months to remove spring devices [38]. Helmet therapy for a year following surgery might be advised [37]. Up until the child is six years old, follow-ups at 3-, 6-, and 12 months are essential for tracking progress [40]. One study offered a comprehensive long-term 3D CT follow-up after endoscopic sagittal craniosynostosis repair. The results indicate that this minimally invasive surgical technique not only successfully addresses the immediate structural and functional issues associated with sagittal craniosynostosis, but also sustains cranial shape normalization and growth over the long term. The study validates the endoscopic technique as a feasible and advantageous solution to this problem, emphasizing the procedure’s ability to minimize the need for more invasive treatments while preserving good results in cranial development [41].

Fronto-orbital advancement (FOA) with cranial vault remodeling: Performed at approximately 9-12 months of age for cases of late-presented trigonocephaly (metopic synostosis), anterior plagiocephaly (unilateral coronal synostosis), and anterior brachycephaly (complete coronal), where the fusion occurs in the frontal region [42]. It involves frontal bones and orbital bandeau being reshaped, the forehead and orbits being advanced forward, and the skull bones being remodeled [40]. A bicoronal zigzag incision is made to execute the procedure. The precise procedures differ according to each person’s anatomy and level of craniosynostosis. While remodeling the bandeau in trigonocephaly, splitting the bandeau and placement of an 8-10 mm interposition graft obtained from the visible parietal bone is achieved [3]. Patients may have edema and discomfort after surgery; these side effects can be controlled with painkillers and close observation. Appointments for follow-up are crucial for tracking healing and guaranteeing the best results [43].

Posterior cranial vault distraction osteogenesis (PCVDO): This procedure is used for cases of sagittal, coronal, lambdoid, or multiple suture synostosis. Compared to conventional procedures, it permits considerable bone augmentation with less intrusive surgery. According to recent reports, PCVDO is the first surgical procedure performed on individuals with elevated ICP brought on by craniosynostosis. It can be performed as early as three months of age; the wide range of patient age indicates safety [44]. To stimulate new bone growth, the procedure entails creating incisions in the skull bones and progressively separating them using a distractor device. However, it necessitates repeated operations, specialized care, and long-term follow-up [45]. A 3-5-day hospital stay is required following the 2-3-hour operation. After that, the surgeon will start turning on the distractors one to two times daily over three weeks. Caregivers continue turning on the devices at home throughout the active distraction period. Weekly follow-up by skull X-rays and clinical examination are the most important parts of the procedure [46]. After that, distractors are left in place for a period of solidification of the fresh bone that was formed. A second surgery for the removal of distractors is held approximately three months after the primary procedure [44].

Posterior vault reconstruction: To support proper skull growth, posterior vault reconstruction is a surgical treatment frequently utilized for lambdoid synostosis in infants or young children. To realign or restructure the skull bones and for healthy brain development, it entails making exact incisions in the posterior region of the skull [40]. Early intervention could prevent complications [47]. The timing of the surgical procedure has been advocated during the first few weeks after birth or during the first year of life, preferably prior to 9 months of age [48]. Healing differs, and physical therapy, cranial helmet therapy, and routine follow-ups are possible postoperative care options [49].

Total calvarial remodeling: Complete calvarial remodeling might be required in complicated cases of multi-suture synostosis such as acrocephaly, oxycephaly, and turricephaly. To ensure sufficient bone strength for a safe fixation, the treatment is best carried out after the child is 8-9 months old [50]. To create a more symmetrical and aesthetically beautiful skull shape, this surgery involves osteotomies, bone contouring, repositioning, and grafting. Depending on the patient’s condition and the surgeon’s preference, it can be done in several stages allowing for optimal healing and recovery between procedures [47]. Some surgeons prefer to do it in a single stage to prevent the risk of serious complications [40]. During the postoperative phase, close observation is necessary to check for problems, guarantee adequate healing, and monitor the growth and development of the skull. To evaluate results and resolve any issues, follow-up appointments on a regular basis are required [7].

Conservative Modalities

Positional therapy: This line is an effective treatment method for positional plagiocephaly, a differential diagnosis of true posterior plagiocephaly. To prevent needless referrals and treatment delays for infants, positional plagiocephaly must be distinguished from real posterior plagiocephaly. This can be explained by the fact that, as mentioned previously, positional plagiocephaly is treated by conservative means, while true posterior plagiocephaly or lambdoid craniosynostosis requires surgical intervention [35,51].

Positional forms are primarily corrected conservatively using three major strategies: helmet therapy, physiotherapy, and counter-positioning. No compiled data exists to determine which works best [52]. Positional therapy used to be the mainstay, but its application has resulted in inconsistent outcomes in the past, with about half of the cases failing to recover and needing another line of management [53].

In the following years, the indications of positional therapy became clearer and more substantiated. Jung and Yun have suggested that postural therapy is recommended in infants younger than 4 months and up to 6 months for mild and moderate cases [54]. Currently, the most recent research concludes that repositioning therapy is the main preventive measure against cranial deformities. The relevant literature highlights the need for early screening, and application of preventive measures such as head repositioning and switching the infant's supine position from right to left, especially in the first 2-8 weeks of life when the skull is most susceptible to outside forces [55].

Pediatric physical therapy program: A pediatric physical therapy program is an intervention program combining manipulative techniques and exercises to lessen cranial deformity, musculoskeletal diseases, and postural preference. This line was not considered the first and most effective line in the treatment of deformational plagiocephaly until some major studies proved it, such as the one done in 2019 by Di Chiara and colleagues. The study enrolled 24 infants for whom a standardized pediatric physical therapy intervention program consisting of 16 sessions of physical therapy, each 40 minutes long, once a week, for four months, and the statistical analysis revealed the effectiveness of the protocol as all anthropometric measurements improved [52]. Moreover, the comprehensive systematic review done in 2023 by Blanco-Diaz et al. reached the consensus that a pediatric physical therapy program needs to be the initial course of action for any non-synostotic asymmetry. Manual therapy has been demonstrated to yield the best results among physical treatment techniques; this is especially true when paired with parents, which can have even more positive effects [55].

Molding helmet therapy: Like a bicycle helmet, the helmet often completely encloses the head. To begin with, a 3D laser scan of the patient’s head is used to produce a head model. The purpose of the helmet is to help the head assume a symmetrical and typical shape by fitting the projecting section of the head snugly and leaving space around the flat part. As the patient’s skull expands, the region of the head that protrudes cannot grow as much, but the extra space surrounding the flat part of the head permits the head to grow more toward the relatively less flat part of the head. Parents are told that their children are not allowed to wear the helmet for longer than 20 hours per day. The average length of time for helmet treatment is two to six months [54].

Positional plagiocephaly: When to begin treating cranial abnormalities with helmets is a topic of controversy. Even though cranial growth completion allows for effectiveness up to 12 months of age, it is generally recommended to begin therapy before 6 months for best results [54]. Moreover, when using 3D scan photogrammetry to assess improvement during molding helmet therapy, it was found that infants who started before 7 months of age showed a more noticeable improvement in symmetry [56]. However, regarding cost-effectiveness and potential commercial participation, helmet therapy is seriously questioned. Despite being extremely rare, additional problems with helmet therapy include: (1) insufficient correction due to an ill-fitting helmet; (2) skin damage at the site where the helmet applies pressure; (3) scalp damage and temporary hair loss at the site of helmet application; and (4) contact allergic reaction [54,55]. Consequently, although molding helmet therapy is effective, it has other aspects that need to be considered. That is why the initiation of helmet therapy, according to Jung and Yun, has been linked to the infant’s age and the severity of the condition, such as if the infant is more than 4 months of age with severe plagiocephaly or if the infant is older than 6 months with mild to moderate plagiocephaly but failed to respond to other conservative treatments [54]. In addition, Blanco-Diaz et al. recommended that helmet therapy be used for newborns with moderate to severe plagiocephaly that manifests later in life or for those who continue to have the condition despite receiving conservative treatments [55].

Sagittal craniosynostosis: Traditionally, sagittal craniosynostosis has been seen as a surgical disorder. Early suture reclosure leads to poor outcomes from basic suturectomy. The results have not improved with a wider craniectomy or the use of interposing materials. On the other hand, endoscopic suturectomy combined with the use of a molding helmet after surgery has demonstrated positive outcomes. The authors questioned if wearing the helmet had a major role in the better result because suturectomy patients often rejoin 8-12 weeks after surgery. Sood and associates proved this theory in 2011 when they enrolled four patients diagnosed with sagittal craniosynostosis between 4 and 6 months old. Contrary to conventional wisdom, these cases show that molding helmets can improve skull shape in patients with sagittal craniosynostosis without the need for a suturectomy [57].

Conservative Observation Versus Surgical Intervention

Metopic synostosis: It might be challenging to distinguish between normal closure of the metopic suture with ridging, resulting in midline osseous forehead protrusion, and metopic craniosynostosis [58,59]. Nevertheless, metopic craniosynostosis is treated surgically, while metopic ridging is managed conservatively. Although a physical examination is often sufficient to make the diagnosis, in more challenging situations, a CT scan may provide more information [59]. Furthermore, surgery is the clear recommendation only in the case of the most severe form of metopic craniosynostosis. Experts are divided on whether a patient has mild or moderate metopic craniosynostosis and, thus, if surgery is necessary [5].

Sagittal synostosis, unilateral coronal synostosis, and unilateral lambdoid synostosis: Regarding whether surgery is indicated, there is no discussion, because the aberrant shape of the skull is not likely to improve on its own [5].

The complex management of nonsyndromic craniosynostosis emphasizes the need for early intervention to reduce consequences. Decisions are made depending on characteristics such as type, severity, and age [5]. A recent multicenter study identified previous cases of nonsyndromic craniosynostosis which underwent either surgical or conservative management. Both were evaluated with the Pediatric Quality of Life Inventory (PedsQL). The study indicated no significant difference in quality-of-life results between the two methods, while the impact of untreated craniosynostosis on neurodevelopment and quality of life is questionable. Surgically treated patients are nevertheless more susceptible to physical and cognitive issues than the normal population, indicating the necessity for comprehensive care. Neurocognitive deficits may also persist after surgery [60].

## Conclusions

In conclusion, craniosynostosis is a relatively common complex condition characterized by the premature fusion of cranial sutures that requires early diagnosis and appropriate management. By understanding the classification and various treatment options available, with a multidisciplinary approach involving craniofacial surgeons, pediatricians, and other healthcare professionals, optimal treatment can be achieved to improve the overall outcomes and quality of life for individuals with nonsyndromic craniosynostosis. Early intervention is key to managing craniosynostosis and mitigating its potential long-term effects on the child’s development and overall well-being.

## Appendices

Surgical modalities

**Table 1:** Management Modalities Indications and Timing ICP: intercranial pressure

Surgical Intervention	Indication	Suggested Timing
Endoscopic-Assisted Strip Craniectomy with Postoperative Helmet Therapy	Cases with a single suture, such as sagittal or metopic sutures	Newborns between the ages of 3 and 4 months old
Fronto-orbital Advancement (FOA) with Cranial Vault Remodeling	Late-presented trigonocephaly (metopic synostosis), anterior plagiocephaly (unilateral coronal synostosis), anterior brachycephaly (complete coronal), where the fusion occurs in the frontal region	Approximately 9–12 months of age
Posterior Cranial Vault Distraction Osteogenesis	Cases of sagittal, coronal, lambdoid, or multiple suture synostosis is the 1^st^ line in patients with high ICP secondary to craniosynostosis	As early as 3 months
Posterior Vault Reconstruction	Lambdoid synostosis	Preferable prior to 9 months
Total Calvarial Remodeling	In complicated cases of multi-suture synostosis such as acrocephaly, oxycephaly, and turricephaly	After 8-9 months
Conservative Modalities		
Pediatric Physical Therapy Program	Positional plagiocephaly	1^st^ line at all ages
Positional Therapy	Positional plagiocephaly	Infants younger than 4 months and up to 6 months for mild and moderate cases
Molding Helmet Therapy	Positional Plagiocephaly	Before 6 months
	Sagittal Craniosynostosis	Before 6 months
